# Experimental and Numerical Investigations on the Mechanical Characteristics of Carbon Fiber Sensors

**DOI:** 10.3390/s17092026

**Published:** 2017-09-04

**Authors:** Salem Bashmal, Mohammed Siddiqui, Abul Fazal M. Arif

**Affiliations:** Mechanical Engineering Department, King Fahd University of Petroleum & Minerals (KFUPM), Dhahran 31261, Saudi Arabia; masiddiqui@kfupm.edu.sa (M.S.); afmarif@kfupm.edu.sa (A.F.M.A.)

**Keywords:** strain measurements, piezoresistive materials, health monitoring, carbon fibers, composite materials

## Abstract

Carbon fiber-based materials possess excellent mechanical properties and show linear piezoresistive behavior, which make them good candidate materials for strain measurements. They have the potential to be used as sensors for various applications such as damage detection, stress analysis and monitoring of manufacturing processes and quality. In this paper, carbon fiber sensors are prepared to perform reliable strain measurements. Both experimental and computational studies were carried out on commercially available carbon fibers in order to understand the response of the carbon fiber sensors due to changes in the axial strain. Effects of parameters such as diameter, length, and epoxy-hardener ratio are discussed. The developed numerical model was calibrated using laboratory-based experimental data. The results of the current study show that sensors with shorter lengths have relatively better sensitivity. This is due to the fact short fibers have low initial resistance, which will increase the change of resistance over initial resistance. Carbon fibers with low number of filaments exhibit linear behavior while nonlinear behavior due to transverse resistance is significant in fibers with large number of filaments. This study will allow researchers to predict the behavior of the carbon fiber sensor in real life and it will serve as a basis for designing carbon fiber sensors to be used in different applications.

## 1. Introduction

There is continued research devoted to detecting and identifying damage in structures before they propagate further and cause complete failure. The main purpose is to develop a structural health monitoring (SHM) system that provides an online and real time damage detection via in-situ sensors. The potential benefits of such a system include reducing lifecycle costs, improving reliability and safety, and aiding the design of composite structures. Some of the SHM methods used are acoustic emission, lamb waves, active vibration-based methods, and the strain-based method [1].

Intensive research was conducted on a variety of materials with sensing ability for different applications. The potential of using newly developed smart materials in damage detection is obvious. Strain gauges, fiber optic sensors, piezoelectric sensors, and microelectromechanical systems (MEMS) are among these sensors. Some novel methods aim to embed a network of sensors inside bulk material or between the layers of composite materials so that diagnosis can be done automatically [2,3]. Despite the potential advantages of this method, it is still very costly and time consuming, as the sensors must be embedded during the manufacturing phase of the material.

Moreover, a significant part of the ongoing research is aimed at investigating the applicability of the sensors that are used in the industry to monitor damages in composite materials. A comprehensive review of recent developments on smart fabric sensors can be found in [4]. Among those materials, carbon fiber sensors (CFS) show good promise in structural health monitoring. They fall into the class of piezoresistive materials, and have attracted the attention of researchers due to their ability to act as strain sensors. Carbon fiber-based materials possess excellent mechanical properties and show linear piezoresistive behavior, which make them good candidate materials for strain measurements.

One early research study shedding light on the piezoresistivity of carbon fibers was performed by Wang and Chung [5,6]. It was observed that electrical resistance of carbon fiber epoxy matrix composite changed with the applied strain. Wang and Chung investigated the piezoresistive behavior of short carbon fibers and epoxy resin composites and showed that resins with short fibers have a better response than those with long fibers [5].

Wang et al. illustrated that the piezoresistive behavior of carbon fibers and other similar graphite fibers can be utilized in sensing [7]. The piezoresistivity of carbon fibers was tested under different conditions: single bare fibers, in polymer composites, and in cement composites. It was shown that the bare carbon fiber is not piezoresistive, while embedding short fibers in cement would give maximum gage factor, which is defined as the reversible portion of the change in electrical resistance [5]. The authors investigated different fibers and measured electrical resistance of sense strain variation in composite structures.

Blazewicz et al. [8] studied the piezoresistivity of carbon fibers and graphite fibers with different microstructure parameters and heat treatments. The study showed that the variation in electrical resistance cannot be accurately estimated using geometrical effects only, i.e., change in length and diameter. The piezoresistivity of the fibers is highly dependent on the crystallite sizes and treatment method, which might lead to positive or negative piezoresistivity.

Several studies attempted to use the self-sensing properties of carbon fibers to measure mechanical quantities in carbon composites. Todoroki and Yoshida discussed the monitoring of damages in carbon fiber-reinforced plastic (CFRP) composites by measuring several quantities, like potential change, electrical resistance change and eddy current [9]. The self-sensing capability of carbon fibers in the composite can provide precise measurements by observing the variation in their electrical resistance.

In the work of Huang and Yang, the electrical sensing properties of carbon fiber-reinforced plastic (CFRP) for low strain-level applications were studied [10]. The study observed that carbon microfibers are not completely straight or arranged in parallel, and the total resistivity is affected by the resistance in the transverse direction. Under the application of tension load, the fibers are more concentrated in the transverse direction than when they are stress free. Thus, there is induced non-linearity due to poor alignment and individual breakage of some fibers. The contacts between the fibers is random and cannot be predicted, thus leading to difficulties in modeling the piezoresistive behavior.

However, there are some inherent problems in the resistance measurement of CFRP. For example, the variation of electrical resistance in CFRP composites is highly sensitive to several parameters. Measurements from different studies showed some contradictory results when measured using different probe configurations. Schulte and Baron [11] and other researchers [12,13,14], have reported that, using two-probes, electrical resistance of CFRP laminates in the fiber direction increase with applied tensile load in the fiber direction. However, Chung reported contrary results when the four-probe method was used to measure electric resistance change in the fiber direction during tensile loading [15]. Their results showed a decrease in the piezoresistance (negative gage factor). In the four-probe method, four electrical contacts were made along the sample (two at each end of the specimen). The outer two were used to pass the current, while the inner contacts measured the voltage.

It was found after further studies by the researchers [16] that the carbon electrodes used for the electrical contact had low reliability, causing of the negative piezoresistivity. In carbon paste, electrical contact is attained only at several discrete points with the carbon paste electrodes causing a complicated electrical current path in the specimen. When silver paste was used, positive piezoresistivity was obtained. Additional tests were performed by the authors themselves and they confirmed that poor electrical contact in the four-probe method was the cause of the negative piezoresistivity [17]. Todoroki et al. examined the anisotropy of piezoresistivity in unidirectional single-ply CFRP subject to multiaxial loading. The unsymmetrical piezoresistivity matrix was calculated using the measured piezoresistivity [18]. It was concluded that negative piezoresistivity was mainly caused by fiber misalignment and sparse electrical contact at the electrodes.

Moreover, the self-sensing piezoresistivity in CFRP is relatively low and may not be suitable for high strain-level applications. Several studies attempted to improve the self-sensing behavior by enhancing the properties of the CFRP. Huang and Yang developed a hybrid-CFRP composite that consists of different types of carbon fibers and were able to obtain larger resistance variations. However, the resistance-strain curve has sudden jumps due to fiber breakage and could lead to irreversible signal singularity [10]. Other researchers used nickel nanostrands and embedded them between the layers of CFRP in order to improve the piezoresistivity [19]. However, there are still many challenges to assuring that the modified composite possesses excellent piezoresistive properties without sacrificing any of other desirable properties.

There are several studies that focused on the use of carbon fiber tows and embedded them in structures for strain measurements and damage detection. In the work of Horoschenkoff et al., the authors investigated the piezoresistivity of commercially available carbon fibers [20]. The fibers were tested to find the influence of relatively high strain levels laminate micro-cracks on the carbon fibers resistance. Micro-cracks were detected using carbon fibers which showed the potential to use them as a sensor for composite materials. The study used carbon fibers with 1 K filaments, which exhibit a linear relation between strain and change in resistance. However, nonlinear behavior was observed due to damage of the electrical connections for high strain level. Further investigations are required on fibers with larger diameters since they can withstand larger strain but suffer from nonlinearity at a lower strain.

In a subsequent study, Horoschenkoff et al. characterized the piezoresistivity of carbon fiber sensors in two industrial applications: a computer tomography (CT) scanner, and pressure vessels [21]. For the CT scanner application, the authors applied the carbon fiber sensor into a carbon fiber-reinforced plastic table of a computer tomography scanner [22]. Unlike metallic materials, carbon fiber is a nonmetal and does not interfere with X-rays. The metal wiring cannot be in the table because they will interfere with the X-rays; therefore, U-shaped carbon fiber sensors were used and the slope of the beam was found using integral strain measurement method.

Further investigations were performed by the authors in order to monitor crack density using a carbon fiber sensor [23]. They also carried out experiments on glass fiber-reinforced polymer specimens and pressure vessels. Analytical and numerical analyses were performed and a good correlation was shown between the crack density and strain measured by the carbon fiber sensor.

In the study of Huang and Wu, low-level strains were measured using long carbon fibers of 12 K filaments [24]. Static and dynamic uniaxial tensions were applied to the steel specimen which has CFS sensors embedded in it. Static and dynamic response measured using carbon fiber sensors agreed very well with those obtained from regular strain gauges. The work’s focus was on presenting a signal processing method facilitating the use of long carbon fibers in damage detection.

More recently, a long-gauge carbon fiber line sensor was introduced in [25] for structural health monitoring and was implemented by Saifeldeen et al. in [26], in conjunction with an auxiliary carbon fiber line sensor to compensate for errors in readings. The lengths of the sensors were 50 cm. The authors showed that the use of two sensors reduced error and showed good linearity under low strain levels. Moreover, the study illustrates that post-tensioning of the sensors can significantly enhance their linearity and cyclic ability.

As seen in previous literature, there is a need for a study that compares the behavior of fibers with different number of filaments in order to facilitate the selection for different applications. Fibers with relatively low diameter tend to follow linear behavior, while those with large diameter exhibit nonlinearity in their behavior. Moreover, the single piezoresistive coefficient is not sufficient to describe the behavior of the fibers. A comparison between those fibers along with the estimation of their piezoresistive properties needs further study.

The current study includes both numerical and experimental tasks in order to investigate the performance of carbon fibers in strain measurements. The investigations were performed on carbon fiber tows that are commercially available in order to assess their performance in measuring strain. A numerical model is developed to investigate the effect of various parameters such as size, diameter and material properties. To calibrate the numerical model, experiments are performed on carbon fibers to measure their performance in real time. The experiments will allow us to assess the ability of carbon fibers in detecting damages caused by various loads. Necessary pretreatment, preparation and installation techniques are discussed, to make them useful for the industry.

## 2. Experimental Work

### 2.1. Preparation of Carbon Fibers

The type of carbon fibers used in the current research were carbon fiber tows or rovings which were based on a commercially available Toray T300b. The choice of Toray T300b was based on its performance in the literature [20,21,25,27]. Each roving contains a bundle of long strand of fibers. The name of the product indicates the number of filaments in the tow. For example, (T300B-3000) refers to the fiber with 3000 filaments (sometimes indicated as 3 K).

Bare carbon fibers cannot be directly used as sensors [7]. The change in resistance observed is solely due to the change in dimensions (diameter and length). Therefore, the fibers have to go through a preparation process that will enhance their piezoresistivity. In this paper, the term “carbon fiber sensor” is used to refer to the fibers that passed through the pretreatment process. This process include the following phases:

- Pre-curing: to stabilize carbon fiber and connect electrical wires for measuring electrical signals
- Curing: curing process 180 °C for 90 min in a furnace.
- Embedding: electrical insulation and fixation patch.

**Figure 1:**
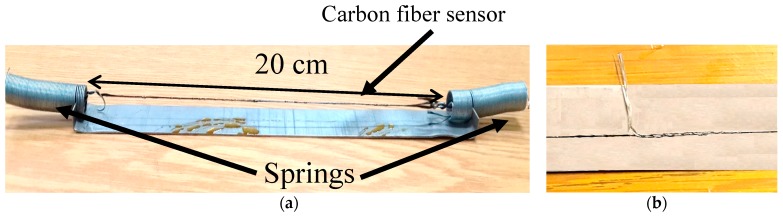
(**a**) Carbon fiber with springs on a metallic bed; (**b**) electrical connections.

In the pre-curing stage, the purpose is to stabilize the carbon fibers and make sure all the individual filaments are aligned together and facing the same direction. This is attained by attaching the ends of the carbon fiber yarn to springs which are fixed on a bed made from metallic strips as shown in Figure 1a.

Moreover, spring elements provide a slight tension on the fibers to ensure that fibers remain straight during the application of the epoxy and heat treatment. The beds along with protruding springs have a total length of 20 cm. The next step is the preparation of electrical connections (see Figure 1b). For this purpose, two short electrical wires were wrapped around the carbon fiber near the end points. Half of the electrical wire is wrapped and the other half is left free. The length between the electrical connections is called the effective length of the sensor.

After the electrical connections are made, the epoxy resin is applied. The epoxy coating is EP301, which is a two-component epoxy resin adhesive. It has a liquid low viscosity consistency and allows a very thin adhesive layer. The fibers are placed in a furnace for 90 min at 180°. For the samples to be used for the tensile testing, the excess length is not removed because that additional length will be used to attach fixation patches which would help secure the sensor in the tensile machine grips.

The preparation steps can be summarized as:

- Fixing the carbon fiber bundle into the bed with springs.
- Making electrical connections to the two ends of the bundle at the designated length.
- Applying epoxy coating on the fibers and allow it to dry.
- Curing the carbon fiber bundle at 180° for 90 min.

### 2.2. Tensile Test Experiment

The principle behind piezoresistive sensors is that their resistance changes when mechanical strain is applied. In order to correlate the resistance with the strains, tensile tests were conducted to observe the variation in electrical resistance with applied strains. In addition, the maximum allowable stress or load can be identified. The tensile tests of the carbon fibers were conducted with various specifications such as length, diameter and treatment of the sensor (see Table 1). This allowed us to characterize the effect of each parameter and gain a better understanding of its effect on sensor performance. Thus, our parametric study consisted of three main parameters; length, width and treatment/epoxy ratio.

The tensile tests were carried out on an INSTRON 3367 machine, The machine can bear a maximum load of 30 kN, a minimum extension speed of 0.005 mm/min and total vertical test space of 1193 mm [28]. The machine has a built-in extensometer that allows the calculation of stresses and strains automatically and obtain the stress–strain curves.

To perform the tensile test, the carbon fiber sensor was mounted by installing grips on the specimen’s ends to prevent slippage. The grips were prepared by using two acrylic pieces bonded together with a strong adhesive. The rate of extension on the tensile machine was set to 1 mm/min which is slow enough to properly record the stress–strain data. However it was reported in the literature that T300 fibers can be regarded as strain rate-insensitive material, so the rate of extension does not have significant effect on stress development [29].

The multimeter is connected to the electrical connections of the sensor and is further connected to a PC for data recording. The data is recorded in time which can then be tallied with the data from the tensile machine to plot graphs of change in resistance versus change in strains.

## 3. Numerical Model

The computational model of the carbon fiber sensor was developed in the COMSOL platform. Each tow or strand of carbon fiber contains small filaments each of 7 μm. Each strand is modeled as a single whole fiber. The material is assumed to be homogenous. The effect of epoxy resin is properly estimated and added to the effective properties of the material, using the results from the experiment. The electrical connections are assumed to be at the ends of the fiber so the voltage is measured from one end to the other.

**Figure 2:**
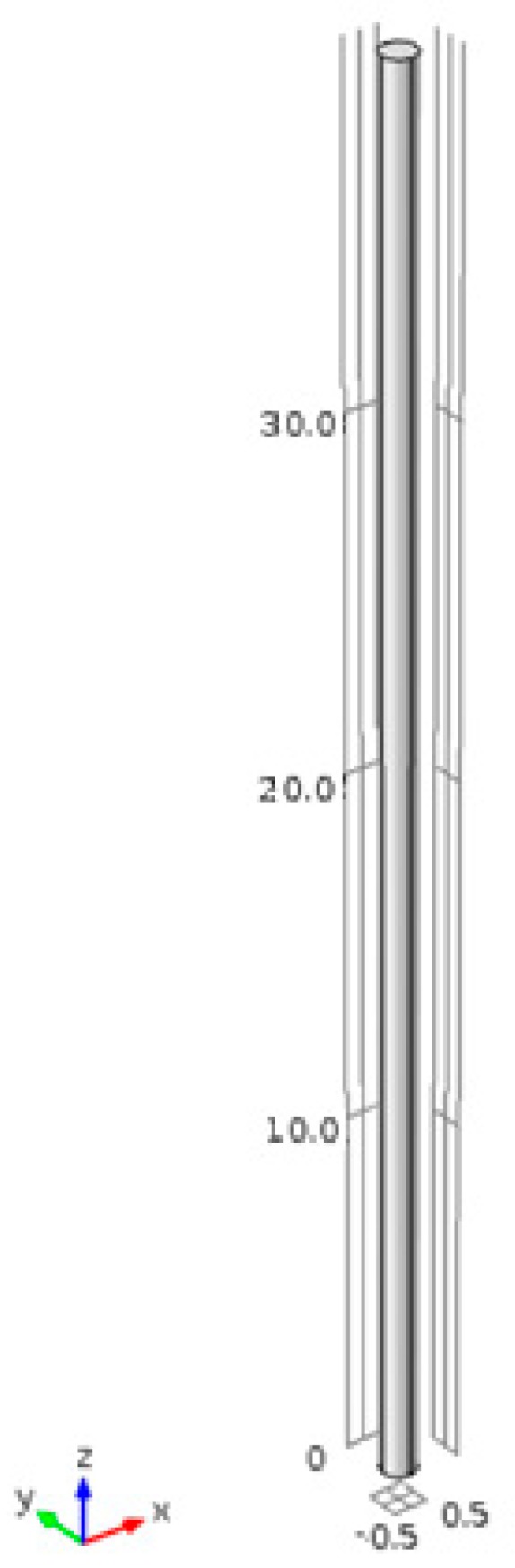
Geometric model of the sensor (dimensions are in mm).

The model of carbon fiber sensor is calibrated using experimental data. The dimensions of the sensor can be varied to the desired values. The size shown in the Figure 2 is of a sensor of length 40 mm and diameter of 1 mm. The lowest cross sectional area was constrained in all directions while axial force was applied to the other end to stretch the sensor. The force applied was increased gradually to obtain relation between change in resistance and change in strains due to the applied forces.

Carbon fibers are anisotropic materials. They have different material properties in different directions. The carbon fibers are considered to be transversely isotropic. The material used in the model is considered to be impregnated with epoxy resin. The relationship between strains and stresses is defined by only five independent constants for transversely isotropic materials. The matrix relating strains to stresses is given as:

(1) $$\left[\begin{array}{c} \varepsilon_{xx}\\ \varepsilon_{yy}\\ \varepsilon_{zz}\\ \varepsilon_{yz}\\ \varepsilon_{zx}\\ \varepsilon_{xy} \end{array}\right]=\left[\begin{array}{cccccc} \frac{1}{E_{x}} & -\frac{\nu_{yx}}{E_{x}} & -\frac{\nu_{xy}}{E_{x}} & 0 & 0 & 0\\ -\frac{\nu_{xy}}{E_{x}} & \frac{1}{E_{x}} & -\frac{\nu_{zy}}{E_{x}} & 0 & 0 & 0\\ -\frac{\nu_{xy}}{E_{x}} & -\frac{\nu_{yz}}{E_{x}} & \frac{1}{E_{x}} & 0 & 0 & 0\\ 0 & 0 & 0 & \frac{1}{2G_{yz}} & 0 & 0\\ 0 & 0 & 0 & 0 & \frac{1}{2G_{xy}} & 0\\ 0 & 0 & 0 & 0 & 0 & \frac{1}{2G_{xy}} \end{array}\right]\left[\begin{array}{c} \sigma_{xx}\\ \sigma_{yy}\\ \sigma_{zz}\\ \sigma_{yz}\\ \sigma_{zx}\\ \sigma_{xy} \end{array}\right]$$

where the five independent constants are: $E_{x}\;\mathrm{and}\;E_{y}$ (*x* and *y* components of elastic modulus), $\;\upsilon_{yx}\;\mathrm{and}\;\upsilon_{zy}$ (shear components of Poisson ratio) and $G_{xy}$ (the component of shear modulus) [30].

The piezoresistive matrix which represents the relationship between changes in resistivity and applied strains is:

(2) $$\left[\begin{array}{c} \Delta_{1}\\ \Delta_{2}\\ \Delta_{3}\\ \Delta_{4}\\ \Delta_{5}\\ \Delta_{6} \end{array}\right]=\left[\begin{array}{cccccc} \Pi_{11} & \Pi_{12} & \Pi_{12} & 0 & 0 & 0\\ \Pi_{12} & \Pi_{11} & \Pi_{12} & 0 & 0 & 0\\ \Pi_{12} & \Pi_{12} & \Pi_{11} & 0 & 0 & 0\\ 0 & 0 & 0 & \Pi_{44} & 0 & 0\\ 0 & 0 & 0 & 0 & \Pi_{44} & 0\\ 0 & 0 & 0 & 0 & 0 & \Pi_{44} \end{array}\right]\left[\begin{array}{c} {Τ}_{1}\\ {Τ}_{2}\\ {Τ}_{3}\\ {Τ}_{4}\\ {Τ}_{5}\\ {Τ}_{6} \end{array}\right]$$

where Δ vector is the change in resistivity, Π is the piezoresistive matrix and T is the applied strain matrix.

**Figure 3:**
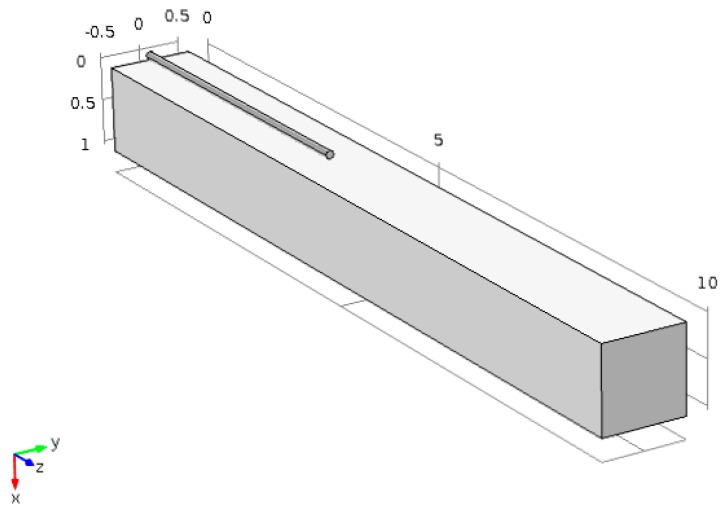
Geometric model of the cantilever beam and carbon fiber sensor.

After the development and validation of the model, the next task is to embed the sensor to a structure and observe the variation of both strain and resistance. A cantilever beam with an embedded carbon fiber sensor on its surface is modeled, as shown in Figure 3. The cantilever provides a good example to test the capability of the carbon fiber to detect strains as the displacements of a cantilever can be calculated using analytical formulas. The sensor is embedded on the upper surface which experienced tensile stress only. The mesh was refined until a convergence was achieved in the displacement results.

## 4. Results and Discussion

Various experiments were performed to test the carbon fiber sensor and a parametric study was carried out to study the effect of parameters that influence our sensors signals. The purpose of the experiments is to study the relation between strains and change in resistance and what parameters have significant effect on the performance.

For the tensile experiments on the carbon fibers, tests were first conducted to observe the stress–strain behavior of the carbon fiber. This will also indicate the fracture point at which the sensor would break. Based on this information, the strain levels at which the carbon fibers operate can be estimated.

**Figure 4:**
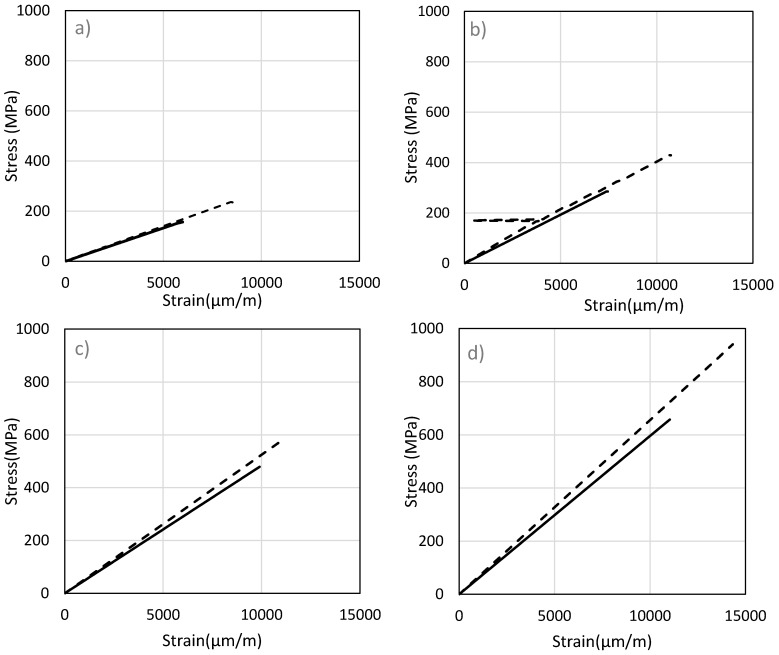
Stress–strain curve of fibers with different number of filaments: (**a**) 1 K, (**b**) 3 K, (**c**) 6 K and (**d**) 12 K; 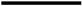: bare fiber and 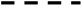: carbon fiber sensor.

For each of the four carbon fiber tows, tensile test is performed with and without the epoxy coating, to observe how the epoxy affects the stress–strain curves. Results of 1 K, 3 K, 6 K and 12 K are shown in Figure 4 It can be observed from the graphs that coating increases the strength of the carbon fiber and makes them able to withstand higher stresses. As the number of fibers increases, the stress needed to break the fibers rises. The modulus of elasticity is affected by the interface between filaments and fiber breakage. Moreover, the broken fiber density is large when the total number of fibers is low [31]. As stated by Wang and Chung, fiber breakage causes a significant reduction in the modulus of the fibers [32]. It can also be noted that the fibers can have maximum strain level of up to 15,000 μm/m for 12 K fibers.

### 4.1. Effect of Diameter on Change in Resistance

In this study, the effect of diameter of the carbon fiber sensor was observed. Four different sample diameters, or number of filaments, were tested: 1 K, 3 K, 6 K and 12 K fibers. All other parameters such as length and epoxy treatment were kept constant so that the variation of the response due to number of filaments can be observed.

**Figure 5:**
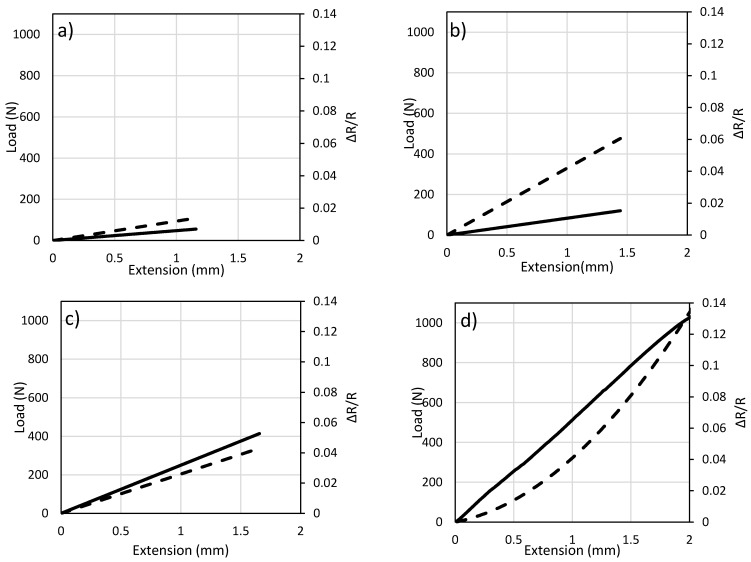
Variation of load and relative resistance vs extension for different number of filaments: (**a**) 1 K, (**b**) 3 K, (**c**) 6 K and (**d**) 12 K; 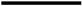: load, and 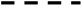: ΔR/R.

The results of 1 K sensor, shown in Figure 5a, illustrate that the 1 K carbon fiber sensor can detect loads up to 60 N. The relationship between changes in resistance/initial resistance (*ΔR/R*) versus extension is linear. Because of low number of fibers, this sensor can bear only up to 60 N with the lowest relative change in resistance among all fibers. Similarly, the variation of resistance versus extension of 3 K, 6 K and 12 K are shown in Figure 5b–d, respectively.

In the plot of 12 K carbon fiber sensor, Figure 5d, the load versus extension is linear. However, the variation of resistance exhibit nonlinear pattern. The nonlinearity may be attributed to wrinkling or slippage due to high number of fibers. However, the main cause of nonlinearity is that the fibers may not be properly treated with epoxy resin. This would lead to transverse resistance and result in nonlinear trend. This nonlinear fashion was also observed in results reported in literature in [9,25].

**Figure 6:**
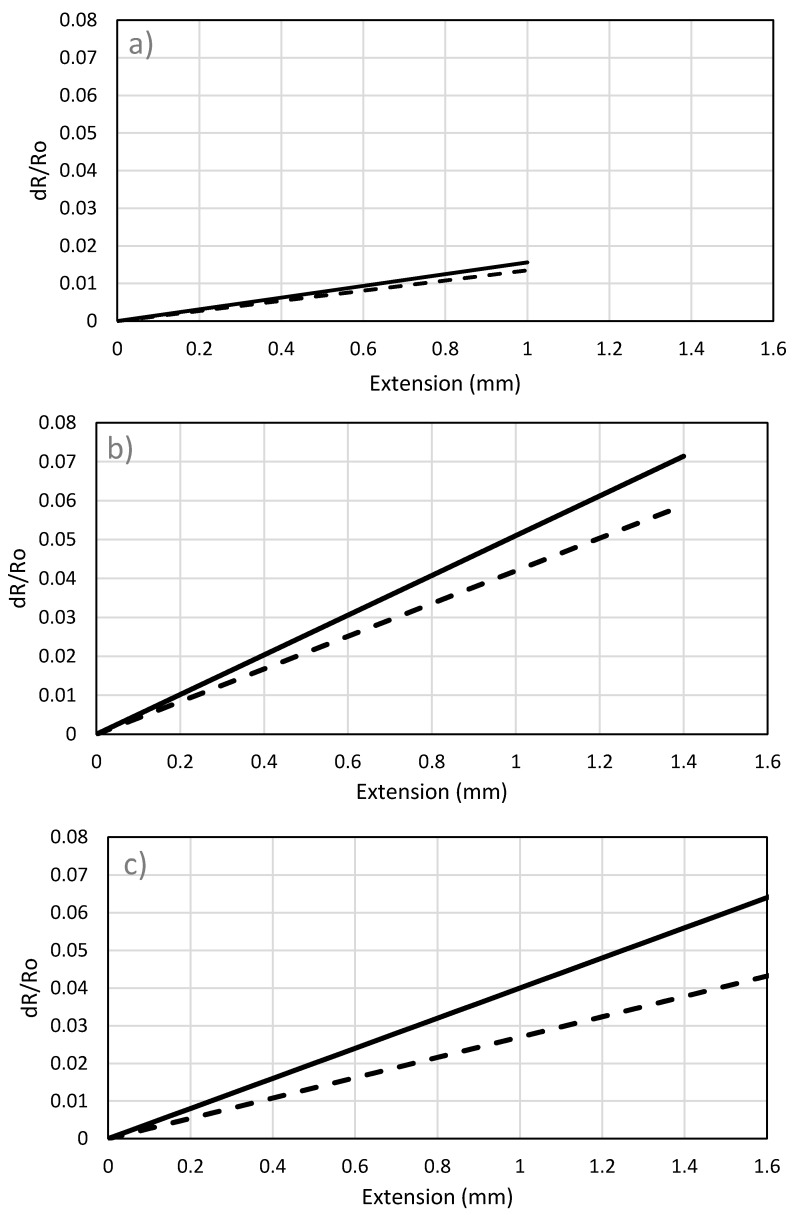
Comparison between analytical and experimental values of: (**a**) 1 K, (**b**) 3 K, (**c**) 6 K; 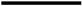: analytical and 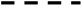: experimental.

The experimental results were verified with the analytical values calculated using the analytical formula:

(3) R=ρL/A

where ρ is the resistivity of the material, *A* is the cross-sectional area and *L* is the length of the filament. The results are shown in Figure 6 where it can be observed that experimental values are slightly lower than the calculated ones. The lower values of the experiment are attributed to the increase in piezoresistance of the fibers. It is noticed that mostly the change in resistance is due to change in dimensions. The results are consistent with the findings of Blazweick et al. who observed that values obtained from experiment are lower than those estimated analytically using the linear equation that governs variations of resistance due to change in geometrical parameters [8].

From Figure 5, it is observed that 1 K fibers have the minimum variation of electrical resistance. Generally, for a low number of filaments (1 K and 3 K) the variation in electrical resistance is due to the change in geometrical parameters (length and diameter) and can be estimated using Equation (3). For large number of filaments (6 K and above) transverse resistance due to the interference between fibers causes the slope of *ΔR/R* to decrease at the low strain levels. The variations of electrical resistance for 6 K and 12 K carbon fibers display similar behavior up to 0.5 mm. The 6 K and 12 K carbon fibers exhibit less variation of *ΔR/R* than the variation associated with the 3 K fibers. As shown in Figure 6c, the deviation from analytical results, obtained using Equation (3), is more significant for the 6 K fibers. This indicates that the change in resistance is not solely attributed to change in dimensions.

While fibers with a large number of filaments (6 K and above) are influenced by the transverse resistance, the 6 K fibers follow linear trend while the 12 K fibers show non-linear behavior after 0.5 mm. In high strain levels, the effect of transverse resistance becomes less significant. Moreover, the increase in *ΔR/R* is attributed to fiber damage which reduces the electrical conductivity [33]. The 6 K fibers are more vulnerable to breakage at high strain levels which leads to sudden failure, while the 12 K fibers experienced gradual fiber breakage before complete failure of the sensor.

Thus, the 1 K and 3 K exhibit a linear trend and can be used for low-level strain applications. The 6 K fibers are not recommended because the resistance variation is lower than the 3 K fibers and they do not provide a higher strain range. The 12 K fibers have the highest range of strains, stresses, and resistance variations, but nonlinearity is inevitable and has to be taken into account.

### 4.2. Effect of Length of Sensor on ΔR/R

In this study, sensors with different lengths were tested and the change of electrical resistance was observed. Three type of fibers were considered: 1 K, 3 K and 6 K. Samples of the 12 K carbon fibers were not included in the current study, as they require further analysis due to the nonlinearity. The other parameters such as diameter and epoxy treatment were kept constant so the response to length variation can be observed.

**Figure 7:**
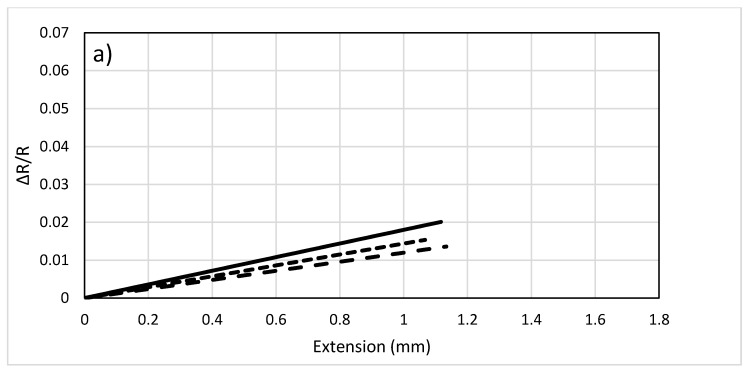

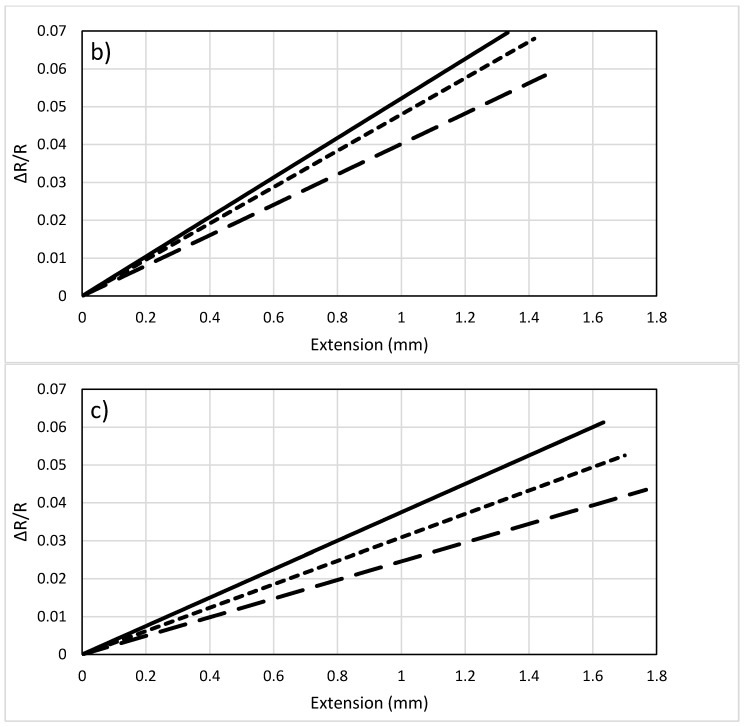
Effect of length on ΔR/Ro for carbon fiber sensors: (**a**) 1 K, (**b**) 3 K, (**c**) 6 K; 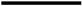: 4 cm, 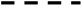: 8 cm, and 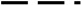: 12 cm.

The plot in Figure 7a represents the change in resistance versus extension for the 1 K sample. It can be observed that as the length increases, the change in resistance decreases slightly indicating that when the length of sensor increases, it becomes less sensitive to the change in strain. Similar trends can be observed in the 3 K and the 6 K carbon fibers, in Figure 7b,c, respectively. Thus, for low strain level, the length of the sensor does not have significant effect on the sensitivity. For measuring high strain level using fibers with large number of filaments, shorter sensors tend to have better sensitivity.

**Figure 8:**
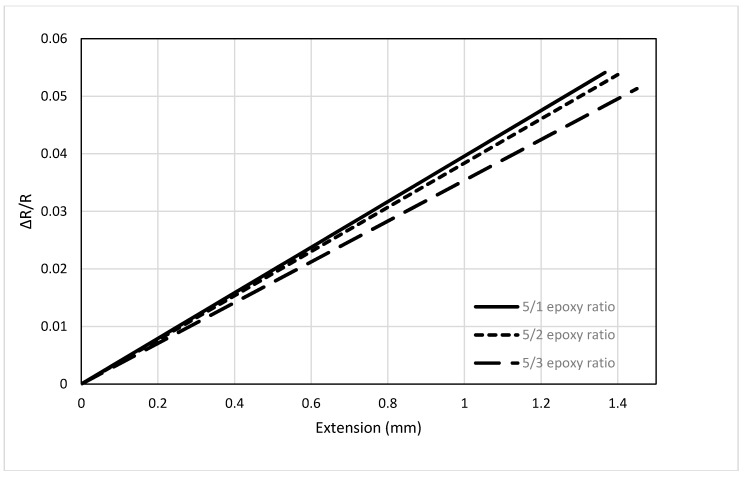
Effect of epoxy treatment on carbon fiber sensor.

Next, the effect of epoxy/hardener ratio during treatment of the carbon fiber on the resistance variation was investigated. Only one fiber diameter can be used to test the effect of treatment and for that 3 K sample was selected because it displayed linear behavior in previous studies. The other parameters such as length and diameter were kept constant so the variation of response due to different epoxy treatment can be observed. Figure 8 shows the results of the study, where 5/1 refers to a ratio of 5:1 between the epoxy and hardener. The figure shows that increasing the ratio of the hardener increases the stiffness of the fibers and causes lower variation of the resistance. However, it can be observed that changing the ratio of epoxy and hardener does not have significant effect on the response of the sensor.

### 4.3. Numerical Results

In this section, results from the computational model of the carbon fiber sensor were presented. Strains were applied by fixing the lower end and having a gradual increase in applied force to the other end. The maximum displacement occurs at the top end, where force is applied. Stress and strain distributions are uniform.

**Figure 9:**
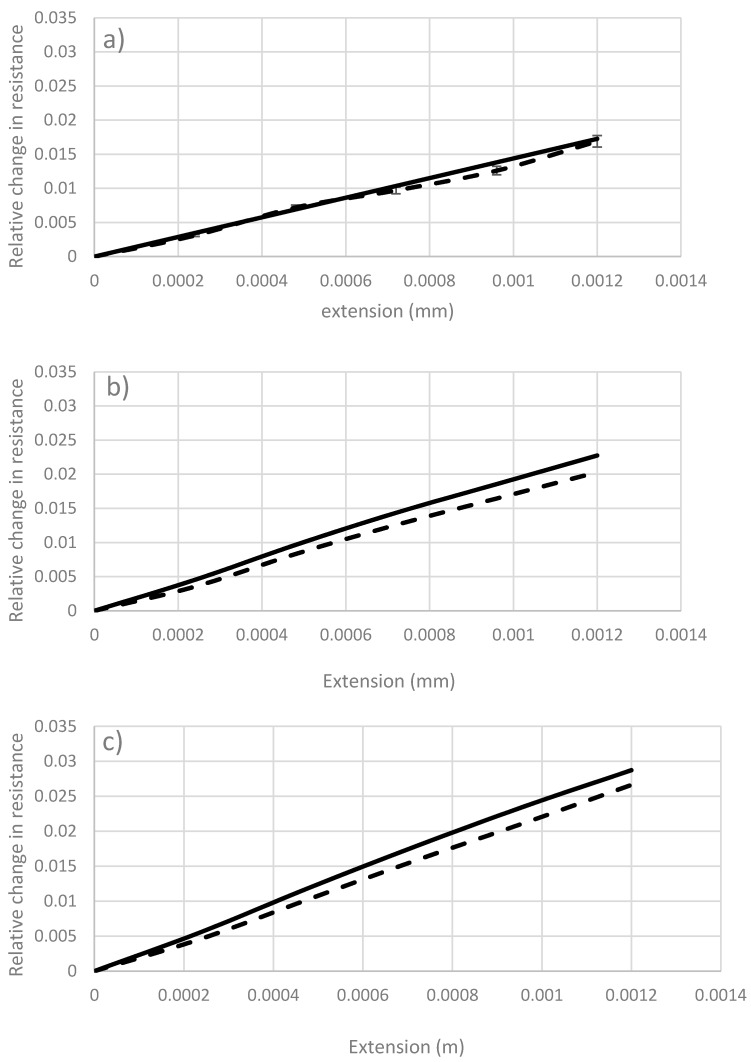
Model validation of computational model with experimental data for 1 K: (**a**) 12 cm, (**b**) 8 cm, (**c**) 4 cm; 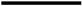: numerical, and 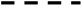: experimental.

The numerical model requires the piezoresistive coefficients to be determined in order to solve the differential equations. Thus, the model was calibrated using the experimental data. Parameter estimation was performed on piezoresistive coefficients until the computational results agreed with the experimental ones, as can be observed in Figure 9a. The piezoresistive coefficients of the carbon fiber sensor obtained after calibrating the model with the experiments are shown in Table 2.

To ensure the accuracy of the estimated coefficients, the model was tested with different lengths: 8 cm and 4 cm of the same model. Since the material is same, the models with different lengths would have same piezoresistive coefficients and the obtained results should match with those obtained from the experimental data. Figure 9b,c show the validation of computational and experimental data for the 1 K fibers of 8 cm and 4 cm, respectively. The results show that the model agrees very well with the results of experiments.

### 4.4. Model Calibration Using Different Piezoresistive Coefficients

The model of carbon fiber sensor was calibrated using experimental data and validated by observing the accuracy of the model when length was changed. The piezoresistive coefficients are shown in Table 2. It can be observed that $\pi_{44}$ have no significant effect on the resistance variation. The parameter $\pi_{11}$ increases by a factor of three between the 1 K and 3 K fibers and then increases sharply for 6 K and 12 K. The parameter $\pi_{12}$ is always negative and its absolute value increases with number of fibers. While these parameters could be used to calibrate the numerical model, further investigation is required in order to draw a conclusion about their physical relevance. The parameters for 12 K assume a linear behavior, while the experimental results are nonlinear. It is, therefore, essential to develop a piezoresistive matrix that is strain-dependent in order to capture the nonlinearity in fibers with large number of filaments.

**Figure 10:**
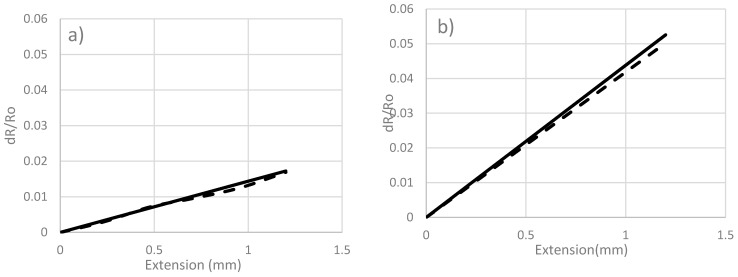

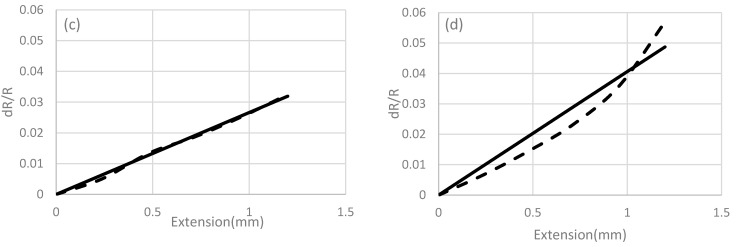
Variation of load and relative resistance vs extension for different number of filaments: (**a**) 1 K, (**b**) 3 K, (**c**) 6 K and (**d**) 12 K; 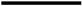: numerical, and 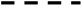: experimental.

Figure 10 shows the response of the model using piezoresistive parameters estimated using the experimental test of the carbon fiber sensors. The numerical model provides a tool for a constitutive relationship of the coefficients as a function of size.

As noted previously, 6 K and 12 K carbon fiber sensors had a non-linear behavior due to several reasons such as transverse resistance and fiber breakages in the experiment. Therefore, when using the linear fitting of the 12 K curve, the results have good comparison but still inaccurate results. The results show significant effect of nonlinearity, which cannot be ignored while comparing carbon fibers with different filaments.

### 4.5. Application of Carbon Fiber Sensor Model

**Figure 11:**
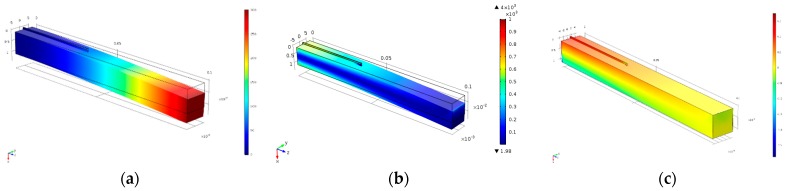
Contour plots of the cantilever beam. (**a**) Displacement; (**b**) Von Mises Stresses; (**c**) Strain distribution.

The 1 K sensor was attached on the upper surface of a cantilever beam close to the fixed end. The beam is made of aluminum with dimensions of 1 cm × 1 cm × 10 cm while the sensor has a length of 4 cm. The displacement contours are shown in Figure 11a. The maximum displacement occurs at the free end but the maximum stresses and strains are developed near the fixed end as shown in Figure 11b,c, respectively.

**Figure 12:**
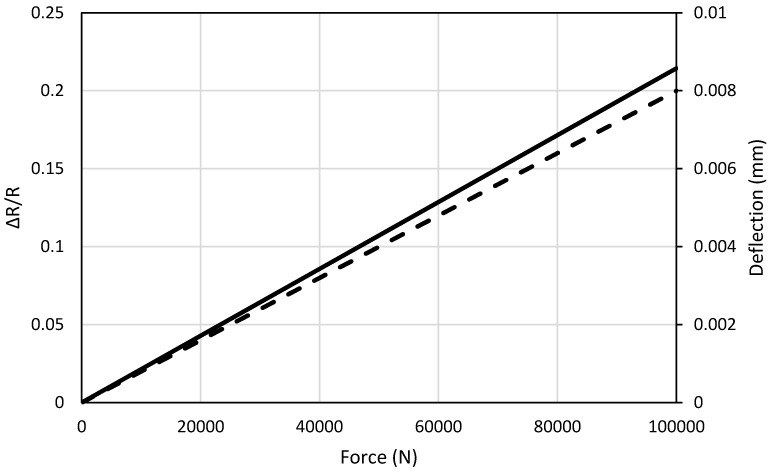
Output of the carbon fiber sensor against applied force and deflection; 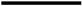: deflection of the tip, and 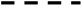: ΔR/R.

The deflection and change in resistance of the sensor in the computational model are plotted against applied force in Figure 12. It is observed that the response of the carbon fiber sensor is linear and increases with the applied force. This application shows that carbon fiber sensor can detect bending in a cantilever beam when embedded on the surface.

## 5. Conclusions

The following conclusions can be drawn from the present study:

- The 1 K and 3 K fibers exhibit a linear trend and can be used for low-level strain applications. The 3 K fibers seem to be more suitable as they can withstand higher stress compared to 1 K, and larger resistance variation, as compared to the 6 K fibers. Moreover, at low-strain level, the sensitivity of 3 K is better than for the 12 K fibers.
- The effect of transverse resistance in the piezoresistivity is significant when the number of fibers is more than 3 K. Fibers with large number of filaments (6 K and above) start to exhibit nonlinear behavior.
- Sensors with shorter lengths have better performance for large number of filaments. For low filaments or a low strain level, the effect of length is not significant. However, long fibers are more susceptible to external influences that might affect their performance.

The numerical model can be used to estimate the change in resistance of carbon fibers of different sizes and lengths. However, more investigations are required in order to capture the nonlinear behavior in fibers with large number of filaments.

## Figures and Tables

**Table 1:** Summary of tensile test experiments.

Carbon Fiber Type	Bare Fibers	With Epoxy
1 K	12 cm	4, 8, and 12 cm
3 K	12 cm	4, 8, and 12 cm *
6 K	12 cm	4, 8, and 12 cm
12 K	12 cm	4, 8, and 12 cm

* Three different epoxy/hardener ratios were tested.

**Table 2:** Piezoresistive coefficients of 1 K, 3 K, 6 K and 12 K carbon fiber sensors.

Carbon Fiber Sensors	$\pi_{11}$ $({\mathrm{MPa}}^{-1}$)	$\pi_{12}$ $({\mathrm{MPa}}^{-1}$)	$\pi_{44}$ $({\mathrm{MPa}}^{-1}$)
1 K	6.53 × $10^{-5}$	−1.0801 × $10^{-5}$	69.05 × $10^{-5}$
3 K	9.154 × $10^{-5}$	−5.0641 × $10^{-5}$	69.05 × $10^{-5}$
6 K	34.55 × $10^{-5}$	−15.7285 × $10^{-5}$	69.05 × $10^{-5}$
12 K	41.79 × $10^{-5}$	−20.6247 × $10^{-5}$	69.05 × $10^{-5}$
